# Serum Amyloid A, Procalcitonin, Tumor Necrosis Factor-*α*, and Interleukin-1*β* Levels in Neonatal Late-Onset Sepsis

**DOI:** 10.1155/2008/737141

**Published:** 2008-11-16

**Authors:** Birsen Ucar, Bilal Yildiz, M. Arif Aksit, Coskun Yarar, Omer Colak, Yildiz Akbay, Ertugrul Colak

**Affiliations:** Department of Pediatrics, Faculty of Medicine, Eskisehir Osmangazi University, 26480 Eskisehir, Turkey

## Abstract

*Background*. Sepsis is an important cause of mortality in newborns. However, a single reliable marker is not available for the diagnosis of neonatal late-onset sepsis (NLS). The aim of this study is to evaluate the value of serum amyloid A (SAA) and procalcitonin (PCT) in the diagnosis and follow-up of NLS. *Methods*. 36 septic and healthy newborns were included in the study. However, SAA, PCT, TNF-*α*, IL-1*β*, and CRP were serially measured on days 0, 4, and 8 in the patients and once in the controls. Töllner's sepsis score (TSS) was calculated for each patient. *Results.* CRP, PCT, and TNF-*α* levels in septic neonates at each study day were significantly higher than in the controls (*P* = .001). SAA and IL-1*β* levels did not differ from healthy neonates. The sensitivity and specificity were 86.8% and 97.2% for PCT, 83.3% and 80.6% for TNF-*α*, 75% and 44.4% for SAA on day 0. *Conclusion*. Present study suggests that CRP seems to be the most helpful indicator and PCT and TNF-*α* may be useful markers for the early diagnosis of NLS. However, SAA, IL-1*β*, and TSS are not reliable markers for the diagnosis and follow-up of NLS.

## 1. INTRODUCTION

Sepsis and septic shock in newborn infants have a high risk of morbidity and mortality. Despite advances in medicine, diagnosis of neonatal sepsis remains as a major challenge. Early clinical signs are nonspecific and the laboratory criteria are also not fully reliable. Warning signs and symptoms are often subtle and can easily be confused with noninfective causes such as apnea, hypothermia, and acute exacerbation of chroniclung disease. So that hematological and biochemical markers such as immature/total neutrophil ratio, platelet count, C-reactive protein (CRP), various cytokines, procalcitonin (PCT), and tumornecrosis factor-*α* (TNF-*α*) have been proposed as being useful indicators for early identification of septic infants [1–4]. Recently, SAA that refers to a group of polymorphic apolipoproteins 12–14 kDa, mainly produced by the liver, has been proposed as a new discriminative marker of bacterial infection [1, 3–6]. But the results of these rare studies are contradictory. In this study, we investigated the value of SAA and procalcitonin levels in the diagnosis and followup of neonatal late-onset sepsis (NLS).

## 2. MATERIAL AND METHODS

Thirty six newborn infants who were diagnosed as having clinical suspected NLS in the neonatal intensive care unit at the medical faculty of Eskisehir Osmangazi University between June 2003 and June 2004 were included in this prospective study. Thirty six healthy newborns who had normal clinical and laboratory findings were included as a control group. They were selected from the neonatal unit or well-baby outpatient clinic. Diagnosis of sepsis was done according to the 2001 International Sepsis Definitions Conference criteria. According to this conference, diagnostic criteria for sepsis in the pediatric population are signs and symptoms of inflammation including arterial hypotension-decreased capillary refill or mottling cardiac index >5.5, significant edema or positive fluid balance, ileus plus infection (documented or suspected) with hyper or hypothermia, tachycardia, and at least one of the following indications of altered organ function: altered mental status, acute oliguria, hypoxemia, increased serum lactate level, or bounding pulses [7]. Laboratory criteria were leukocytosis, leukopenia, immature leukocyte count >10%, and high CRP levels [7].

Töllner's sepsis score (TSS) was calculated for each patient [8]. This scoring system consists of clinical (skin color, body temperature, muscle tonus, breath rate, abdominal distension, imperfect microcirculation, and risk factors) and laboratory (leukocyte and thrombocyte counts, CRP, immature/total neutrophil ratio) parameters. A point was given for each parameter (0, 1, 2, or 3) in respect of their worsening. For example, 0 for normal muscle tonus, 1 for hypotonia, 2 for flask tonus, 0 for normal leukocyte count, 1 for leukocytosis, and 3 for leukopenia. According to this scoring system, patients who have >10 points were determined as having sepsis. Chest radiographs were routinely performed. Hematological and biochemical markers including a complete blood count, differential white cell count, and levels of CRP, TNF-*α*, IL-1*β*, PCT, and SAA were serially measured. The initial (2 mL) blood samples were obtained from patients on day 0 (at the time of sepsis diagnosis). Two further samples were obtained from each patient on days 4 and 8 for follow-up in the patient group. However, only one blood sample (2 mL) was obtained from each healthy control subject.

Serum CRP levels were measured by radio-immuno-assay method (cut-off level: 0.8 mg/dL). TNF-*α* (cut-off level: 1 pg/mL), IL-1*β*, and SAA (cut-off level: 6610 ng/mL) levels were measured by ELISA (BioSource International Immunoassay, BioSource International, Inc., Calif, USA). PCT (cut-off level: 0.8 ng/mL) levels were analyzed by immunoluminometric method (Brahams PCT LUMItest, BRAHAMS-Hennigsdorf, Berlin, Germany). Blood specimens for TNF-*α*, IL-1*β*, PCT, and SAA were immediately immersed in ice and transported to the laboratory for processing. Serum was separated by centrifugation at 4°C and stored in 200 *μ*L aliquots at −72°C until analysis.

The receiver operator characteristic (ROC) method was used to determine the best cut-off point of CRP, TNF-*α*, PCT, and SAA.

The study was approved by the Research Ethics Committee of the Faculty of Medicine, Eskisehir Osmangazi University. Informed consents were obtained from the parents or guardians for all study patients and control subjects.

Statistical analyses were performed using SPSS 10.0. *χ* ^2^, post hoc and Mann Whitney-U tests were used to compare categorical predictors. Also, multivariate analyses were performed for logistic regression analysis. The sensitivity and specificity were calculated for days 0, 4, and 8 to construct the receiver operating characteristic curves for each biochemical marker.

## 3. RESULTS

The demographic characteristics of the study and control groups are summarized in Table 1. There were no significant differences between the two groups for gestational age, sex, birth weight, or Apgar scores at 1 and 5 minutes. Clinical and laboratory findings of the study group are given in Table 2. Hypotonia, changes in body temperature, cut is marmoratus, dyspnoea, and hepatomegaly were dominant findings in patients with sepsis. Platelet counts (Mean ± SEM) and I/T ratios (Mean ± SEM) were higher in patients than in controls (46777.8 ± 10720.6/mm^3^ versus 215000 ± 47600/mm^3^; *P* < .001 and 0.2 ± 0.1 versus 0.002 ± 0.004; *P* < .001; resp., but leukocyte counts (Mean ± SEM) were similar in patients and controls 19294.4 ± 8598/mm^3^ versus 46777.8 ± 10720.6/mm^3^; *P* > .05).

During the study period, 28 (77.8%) patients were treated with mechanical ventilation. Eight out of these 28 (28.6%) patients had respiratory dystress syndrome and were treated with surfactant. Thirty of 36 (83.3%) patients were given oxygen therapy. None of the patients had been treated with pre-/postnatal antibiotics or steroids and none of them had been operated prior to the inclusion to the study. Also none of the patients had a central line.

The ratio of blood culture-proven septic patients among the study group was 72.2%. Klebsiella pneumonia was the most prevalent microorganism in the septic patients.

**Figure 1:**
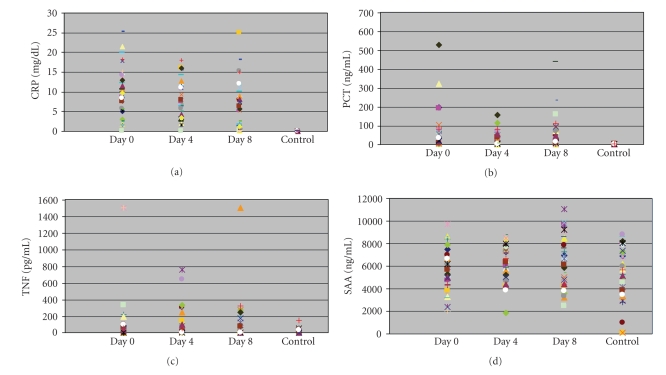
Serum levels of CRP, PCT, TNF-*α*, and SAA on days 0, 4, and 8.

Individual serum levels of PCT, SAA, CRP, TNF-*α*, and IL-1*β* in the whole patient group and control group on days 0, 4, and 8 are shown in Figure 1. However, Table 3 demonstrates serum levels of acute phase reactants in the patients with proven sepsis and controls. The serum levels of CRP, PCT, and TNF-*α* in the whole patient group on day 0 were found to be significantly greater than the controls (Figure 1, *P* = .001). Also, serum levels of CRP, PCT, and TNF-*α* in the patients with proven sepsis on day 0 were found to be significantly higher than in controls. These high levels were continued on days 4 and 8. On the other hand, serum levels of IL-1*β* were not higher in the patient group on days 0, 4, and 8 than the control group. The mean levels of SAA of the study group were found to be greater than that of the control group but the difference was not statistically significant (*P* > .05).

While PCT levels on day 0 were significantly higher than on day 4 (*P* < .05), there were no significant differences between PCT levels on day 4 and day 8 or day 0 and day 8 (*P* > .05). TNF-*α* and SAA levels did not change statistically during the study period (*P* > .05). CRP levels on day 0 were significantly higher than on day 4 and day 8 (*P* = .05 and *P* = .01, resp.). However, CRP levels on day 4 and day 8 were similar (*P* > .05).

TSS did not correlate with the serum levels of PCT, SAA, CRP, TNF-*α*, IL-1*β*, or hematological values including I/T ratio, leukocyte, and thrombocyte counts (*P* > .05). Also, the serum levels of PCT, SAA, CRP, TNF-*α*, or IL-1*β* did not correlate with platelet counts (*P* > .05).

The serum levels of CRP negatively correlated with the levels of SAA and positively correlated with TNF-*α* on day 0 (*r* = −0.532, *P* < .001; *r* = 0.393, *P* < .05, resp.) and with the levels of SAA on day 4 (*r* = −0.481, *P* < .01). In addition, the levels of SAA on day 0 positively correlated with the ratio of I/T (*r* = 0.40, *P* < .05).

Multiple regression analysis showed that the most important factor which influenced the levels of CRP on days 0, 4, and 8 was TNF-*α* level (*r* = 0.896, *P* = .001; *r* = 0.621, *P* < .05; *r* = 0.634, *P* < .01, resp.). The sensitivity and specificity of PCT, SAA, CRP, and TNF-*α* for determining sepsis are summarized in Table 4. The sensitivity and specificity of the acute phase reactants on day 0 were CRP (97.2%; 95% CI: 85.4–99.5 and 100%; 95% CI: 100.0-100.0), PCT (86.1%; 95% CI: 70.5–95.3 and 97.2%; 95% CI: 85.4–99.5), TNF-*α* (83.3%; 95% CI: 67.2–93.6 and 80.6%; 95% CI: 64.0–91.8), SAA (75%; 95% CI: 57.8–87.9 and 44.4%; 95% CI: 27.9–61.9, resp.). Based on these analyses, the most sensitive parameter was CRP on day 0. Other sensitive parameters were PCT, TNF-*α*, and SAA, respectively, on day 0.

**Figure 2:**
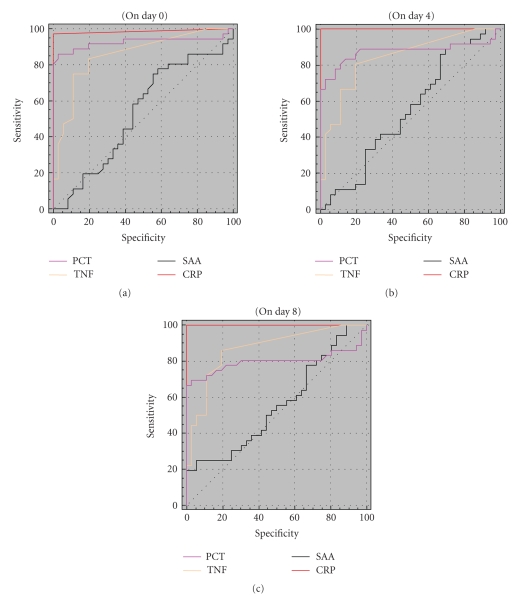
ROC curves and cut-off levels of CRP, PCT, TNF-*α*, and SAA.

The ROC curves of PCT, SAA, CRP, and TNF-*α* are seen in Figure 2.

Three patients (8.3%) died due to systemic inflammatory response syndrome. One of them had hydrops fetalis and two others had respiratory dystress syndrome and also all these three patients had disseminated intravascular coagulation (DIC). Interestingly, we did not find any significant increase in the levels of TNF-*α* and SAA in this patients in comparison to those who survived, but high levels of PCT were found in these patients on days 0, 4, and 8 than the control group (90 ± 7.8/1.5 ± 0.1; 42.2 ± 8.3/1.5 ± 0.1; 56.3 ± 7.2/1.5 ± 0.1, resp., *P* < .001).

## 4. DISCUSSION

Culture-proven bacterial infections in newborn infants are associated with substantial morbidity and mortality. However, a single reliable biochemical marker is not available for the diagnosis of neonatal sepsis. SAA has been proposed for early diagnosis of NLS [4, 5, 9, 10]. Arnon et al. reported that SAA had an overall better diagnostic accuracy for predicting early onset sepsis than CRP (sensitivity (96% versus 30%), specificity (95% versus 98%)) [4]. Also, they found that SAA was a useful inflammatory marker during late-onset sepsis in preterm infants [5]. In contrast to these studies in our study, SAA levels were greater in patients with late-onset neonatal sepsis than in the control group on days 0, 4, and 8; but this difference was not statistically significant (*P* > .05). These results may explain defective IL-1*β* production in newborn. In fact, it has been reported that SAA gene transcription is induced mainly by glucocorticoids, IL-1, IL-6, and TNF-*α* [11]. However, Gabay et al. [12] reported that IL-1 and IL-6 but not TNF-*α* could induce SAA protein synthesis in human hepatocytes and that IL-6 was the most effective inducer but synergy with either IL-1 or TNF-*α* was not seen [12]. Our experience showed that IL-1 does not increase in septic newborns and TNF-*α* seems not to influence the production of SAA in septic newborns. Therefore, the production of SAA may not be adequately stimulated in newborn. Like IL-1, we do not propose the use of SAA as an acute phase reactant in NLS.

Although, in recent years, several new markers of infection have been investigated, some studies suggested that CRP remains to be the best diagnostic test for neonatal sepsis. Especially serial measurements of CRP are highly specific and sensitive in newborn sepsis [12–16]. Also, our results showed that CRP is the best sensitive and specific acute phase reactant for diagnosis of NLS.

Our data indicated that the plasma concentrations of IL-1*β* in infected infants were remarkably low and IL-1*β* was a less satisfactory marker. It seems that the monocytes of newborn infants may be unable to secrete adequate IL-1*β* and prostaglandin E_2_ (fetal or maternal) and IL-6 may suppress IL-1*β* and TNF-*α* production in infections [17–20]. Therefore, we do not propose the use of IL-1*β* as an acute phase reactant in NLS.

TNF-*α* is one of the primary agents which sets in motion the exaggerated cellular, metabolic, and vascular responses of sepsis [21–23]. Its usefulness as a diagnostic marker has not been found to be as good as either SAA or IL-1 in other studies and the sensitivity and specificity have been controversial in neonatal sepsis [13, 17, 24]. In one study, the sensitivity and specificity were found to be 87.9% and 43%, respectively, with high positive and negative predictive values [17]. In our study, it was found that the sensitivity and specificity of TNF-*α* were 83.3% and 80.6%, respectively, on day 0. We found that the levels of TNF-*α* positively correlated with the serum levels of CRP on day 0. So that TNF-*α* is also a useful marker with high sensitivity and specificity for the detection of NLS.

A few new of markers including PCT seem to be promising as a diagnostic test for sepsis [3, 6, 25–29]. Some studies found that the sensitivity of PCT is low (70%–80%) to rule out sepsis at birth [25, 28]. Indeed, Enguix et al. [6] reported that PCT, CRP, and SAA are similar diagnostic markers of sepsis in critically ill neonates. In contrast to these reports, in our study, the order of the markers according to sensitivity and specificity for optimum prediction of neonatal sepsis is CRP > PCT > TNF-*α* > SAA at the time of diagnosis. Therefore, we found that PCT was the second useful diagnostic marker in sepsis after CRP.

Our study confirmed previous findings that neonates with bacterial sepsis have reduced thrombocyte count, high I/T ratio and presence of toxic granulation in granulocytes [28, 29], but leukocyte counts were not found to be increased in our study. Also we found that there was not any correlation between TSS and acute phase reactants or hematological parameters. Caksen et al. [30] reported that there was not a significant difference for leukocyte counts, cytokine levels, and TSS between the blood culture-positive and -negative groups in septic newborns. We consider that Töllner sepsis score is itself not helpful in early diagnosis of neonatal bacterial infections.

In conclusion, our data indicate that CRP is the best reliable marker of inflammation for the diagnosis of NLS. PCT may also be used as a sensitive and specific diagnostic marker in NLS. The SAA did not increase in early phase in our patients, therefore it cannot be used alone for the diagnosis and followup of NLS. Also, TSS is not beneficial in late-neonatal sepsis.

## Figures and Tables

**Table 1:** Demographic findings in patients and control subjects*.

	Sex		Birth weight	Delivery		Age		5′Apgar

	Girls	Boys	(mean ± SD)	SVD	C/S	Gestational	Post natal	score
	*n* (%)	*n* (%)	(g)	*n* (%)	*n* (%)	(mean ± SD) (week)	(mean ± SD) (day)	mean
Patients group	13 (36.1)	23 (63.8)	2281.9 ± 127.1	18 (50)	18 (50)	34.5 ± 0.5	6.9 ± 0.6	9.2
Control group	12 (33.3)	24 (66.6)	2202.1 ± 122.6	20 (56)	16 (44)	33.4 ± 0.4	8 ± 0.8	8.9

SVD: Spontan vaginal delivery; C/S: caesarean section.* *P* > .05.

**Table 2:** Clinical and laboratory findings in patients.

	*n*	%
A. Clinical parameters		

Depressed newborn reflexes	30	83.3
Hypotonia	31	86.1
Change of body temperature	26	72.2
* * * *– High (>38°C)	19	52.8
* * * *– Low (<36°C)	7	19.4
Dyspnoea	26	72.2
Cut is marmoratus	23	63.9
Hepatomegaly	21	58.3
Central cyanosis	20	55.6
Apnea	18	50
Jaundice	15	41.7
Convulsion	8	22.2
Lethargy	5	13.9
Sclerema	5	13.9

B. Laboratory parameters		

Disseminated intravascular coagulation (DIC)	5	13.9
Toxic granulation	33	91.7
High I/T ratio	19	52.8
Blood culture	26	72.2
* * * *– Coagulase-negative staphylococci	2	5.6
* * * *– Staphylococcus aureus	4	11.1
* * * *– Klebsiella pneumoniae	13	36.1
* * * *– Acinetobacter SPP	2	5.6
* * * *– Pseudomonas aeruginosa	1	2.8
* * * *– Enterobacter cloaca	2	5.6
* * * *– Candida tropicalis	1	2.8
* * * *– Streptococcus viridans	1	2.8

Platelet count (mean ± SEM, per mm^3^)	46777.8 ± 10720.6	

Leukocyte count (mean ± SEM, per mm^3^)	19294.4 ± 8598	

**Table 3:** Serum levels of acute phase reactants in newborns with culture-proven sepsis and controls (mean ± SD).

	Patients			Controls
	Day 0	Day 4	Day 8	
Procalcitonin (ng/mL)	89.7 ± 30.3***	50.7 ± 16.8***	62.7 ± 18.7****	1.5 ± 0.1
TNF-*α* (pg/mL)	161.9 ± 78.4*	86.7 ± 28.6***	144.8 ± 58.2**	9.3 ± 4.4
IL-1*β* (pg/mL)	<1	<1	<1	<1
SAA (ng/mL)	5529.6 ± 375.5	5671.5 ± 367	5643.5 ± 436.2	5461.1 ± 407.5
CRP (mg/dL)	10.4 ± 1.1***	8.4 ± 1***	7.3 ± 1.1***	0.8 ± 0.1

*P < .05; **P < .01; ***P < .001; ****P < .0001.

**Table 4:** The sensitivity and specificity of acute phase reactants.

	Sensitivity (%)			Specificity (%)		
	Day 0	Day 4	Day 8	Day 0	Day 4	Day 8
Procalcitonin	86.1	83.3	69.4	97.2	86.1	97.2
TNF-*α*	83.3	80.6	86.1	80.6	80.6	80.6
CRP	97.2	100.0	100.0	100.0	100.0	100.0
SAA	75.0	86.1	19.4	44.4	33.3	100.0
